# Changes in Plant Rhizosphere Microbial Communities under Different Vegetation Restoration Patterns in Karst and Non-karst Ecosystems

**DOI:** 10.1038/s41598-019-44985-8

**Published:** 2019-06-19

**Authors:** Zhouzhou Fan, Shuyu Lu, Shuang Liu, Hui Guo, Tao Wang, Jinxing Zhou, Xiawei Peng

**Affiliations:** 1 College of Biological Sciences and Technology, Beijing Forestry University, Beijing, 100083 China; 2 Department of Biology, Microbial Ecology Group, Lund University, Lund, Sweden; 3 School of Soil and Water Conservation, Beijing Forestry University, Beijing, 100083 China

**Keywords:** Soil microbiology, Forest ecology

## Abstract

Understanding how patterns of recovery and geological conditions affect microbial communities is important for determining the stability of karst ecosystems. Here, we investigated the diversity and composition of microorganisms in karst and non-karst environments under natural restoration and artificial rehabilitation conditions. The results showed no significant differences in soil microbial diversity, but the microbial communities associated with geological conditions and tree species differed significantly. Variation partitioning analysis (VPA) showed that a total of 77.3% of the variation in bacteria and a total of 69.3% of the variation in fungi could be explained by vegetation type and geological background. There were significant differences in six bacterial classes (Actinobacteria, Alphaproteobacteria, Ktedonobacteria, TK10, Gammaproteobacteria, and Anaerolineae) and nine fungal classes (Eurotiomycetes, Agaricomycetes, unclassified _p_Ascomycota, Sordariomycetes, Tremellomycetes, norank_k_Fungi, Pezizomycetes, Leotiomycetes and Archaeorhizomycetes) among the soils collected from six plots. A Spearman correlation heatmap showed that the microbial community was affected by the major soil properties. Principal coordinates analysis indicated that the microbial community of *Pinus yunnanensis* in the artificial forest, which was established for the protection of the environment was most similar to that in the natural secondary forest in the karst ecosystem. These findings further our understanding of microbial responses to vegetation restoration and geological conditions.

## Introduction

Karst landscapes account for approximately 15% of the world’s total land area^1^ and are mostly composed of calcium carbonate rocks containing large quantities of carbonate minerals. The weathering of carbonate rocks in the karst environment influences the geochemical composition of the soils, atmosphere, and organisms, and the transfer of matter and energy among them^2^. The desertification of rocky karst terrain leads to land degradation involving significant soil erosion, extensive exposure of bedrock, and the appearance of a desert-like landscape in which it is difficult for vegetation to recover^3^. Previous studies have discussed the reasons for this desertification process in karst ecosystems. These include anthropogenic disturbances due to pressure from increasing population sizes and overuse of land, as well as natural factors such as precipitation concentration and low vegetation coverage^4,5^.

In an attempt to reduce the rapid degradation of karst ecosystems, initiatives that focus on the recovery of vegetation have been widely implemented because they can improve soil nutrient conditions and other environmental factors^6^. In recent decades, both natural restoration and artificial rehabilitation have been applied to severely degraded lands. Artificial rehabilitation is widely used for reforestation due to its rapid growth and economic value. Natural revegetation does not interfere with natural ecosystems and increases the spatial heterogeneity of soil conditions^7^. Nilsson *et al*.^8^ suggested a comprehensive management approach based on a recovery model that combines natural and artificial forestation in karst ecosystems. Thus, the selection of suitable tree species for artificial rehabilitation would be useful for improving the ecological value of the forest.

Plant species recruit different microbial species into their root phenes from soil for mutualistic benefits^9–11^. Indigenous microbial communities in soil are fundamental for healthy ecosystem function, owing to their role in mediating the circulation of various important materials, such as soil organic carbon and nitrogen^12–14^. Berg *et al*.^15^ analyzed soil stratification differences between fungal and bacterial communities and found that the distribution of the two communities varied depending on the tree species. Moynahan *et al*.^16^ reported that revegetation projects rely heavily on regeneration of the microbial community. The soil bacterial community changed constantly with plant development and root growth, depending on the history of co-evolution among plants and soil microbial communities, to meet nutritional or physiological requirements. This indicates that the relationship between microorganisms and plants is mutually beneficial^17^. In addition, changes in soil factors can influence the composition of the microbial community. Rousk *et al*.^18^ studied the influence of pH on microbial community composition and found that the pH range for optimal growth of bacteria was narrower than that for fungi. Smith *et al*.^19^ showed that soil organic matter (OM) aggregate pools played an important role in the spatial distribution of fungal- and bacterial-dominated communities and influenced microbial distribution and richness by promoting deterministic processes within the karst system.

Several studies on degraded karst ecosystems have focused on changes in soil quality^20^ and microbial community^19^ during the recovery of vegetation. Cao *et al*.^21^ investigated the soil microbial communities in plantations and reported that soil microbial biomass and community composition varied according to the environment of the sampling site. Li and Shao^22^ reported that soil physical properties on degraded farmland improved (i.e., soil bulk density decreased and soil aggregate stability and saturated hydraulic conductivity increased) with the succession of natural vegetation. In addition, microbes affect the stability of carbonate rocks and the rate of karstification^2^. Hu and Guo^23^ thought that the capacity for using carbon sources and the functional diversity of soil microbial communities was stronger in natural restoration forests than in artificially rehabilitated land. Chen *et al*.^24^ reported that microbial resource limitation was different and that modeled decomposition and respiration rates were significantly higher in karst forest than in non-karst forest. However, the difference of recovery pattern (natural restoration or artificial rehabilitation) on microbial communities in relatively fragile karst ecosystems and in non-karst ecosystems remains poorly understood in Jianshui, China.

Thus, in this study, we investigated the (i) recovery patterns, (ii) geological conditions, and (iii) influence of edaphic properties on microbial communities in karst and non-karst ecosystems. We hypothesized that (i) there would be greater microbial diversity in the non-karst system than in the fragile karst system within the same forest type and (ii) that changes of recovery pattern (natural restoration or artificial rehabilitation) would impact the rhizosphere of soil microbial communities. We expect our findings to help provide a basis for more effective management of degraded karst ecosystems from the perspective of microorganisms.

## Results

### Physicochemical soil properties

Soil physiochemical properties varied among sites (Table 1) and were influenced by geological conditions (karst vs. non-karst) and forest type. Karst soils beneath the same plant species generally had higher water, total nitrogen (TN), available potassium (AK), pH, and, in particular, organic matter (OM) contents than non-karst soils, while non-karst soils had higher electrical conductivity (EC) and total phosphorus (TP), with the exception of samples collected from sites in secondary forest. Compared to the artificial forests, under the same geological conditions, the secondary forest soils were richer in OM, AK, and TP and had higher pH values. The highest OM concentrations in karst and non-karst soils were 127.27 and 55.08 g·kg^−1^, respectively. All of the soil samples were acidic, with pH values of 3.92–6.92. Under the same vegetation conditions, the pH values of the karst soils were higher than those of the non-karst soils. The EC of the soils varied from 2.62 to 4.65 µS·cm^−1^, and was correlated with the salinity of the soil. Our results indicated that the soil physiochemical properties were affected by the combination of geological environment and vegetation type.

**Table 1:** Physicochemical properties of tested soils.

Samples	Water Content (%)	AK (mg·kg^−1^)	TN (g·kg^−1^)	TP (g·kg^−1^)	OM (g·kg^−1^)	pH	EC (μs·cm^−1^)
KE	24.80 ± 0.51b	134.20 ± 3.41c	0.77 ± 0.22b	0.33 ± 0.06c	94.35 ± 0.98b	5.41 ± 0.15c	2.62 ± 0.11c
NE	9.99 ± 0.31d	127.13 ± 1.55d	0.54 ± 0.04b	1.51 ± 0.03bc	31.02 ± 0.70e	5.20 ± 0.16c	2.9 ± 0.08c
KP	21.76 ± 0.45c	119.50 ± 2.31e	3.33 ± 0.16a	0.30 ± 0.08c	63.95 ± 0.78c	6.35 ± 0.26b	2.75 ± 0.21c
NP	11.56 ± 0.21d	43.40 ± 0.98f	0.44 ± 0.26b	0.34 ± 0.11c	15.86 ± 0.12f	3.92 ± 0.02d	3.49 ± 0.01b
KS	26.41 ± 1.44a	264.70 ± 1.47a	1.61 ± 0.05ab	3.32 ± 0.32a	127.27 ± 0.40a	6.92 ± 0.29a	4.65 ± 0.41a
NS	21.27 ± 0.26c	185.33 ± 2.75b	0.94 ± 0.03b	2.65 ± 0.03ab	55.08 ± 0.70d	5.54 ± 0.09c	3.01 ± 0.34c

KE: *E. robusta* in karst areas; NE: *E. robusta* in non karst areas; KP: *P. yunnanensis* in karst areas; NP: *P. yunnanensis* in non karst areas; KS: secondary forest in karst areas; NS: secondary forest in non karst areas. AK: available potassium; TN: total nitrogen; TP: total phosphorus; OM: organic matter; EC: electrical conductivity. Data is reported as mean ± standard error (n = 3); Means with different letters within a column are significantly different at *P* < 0.05.

### Distribution of taxa and phylotypes

The rarefaction curves for both the bacterial and fungal communities indicated that the variation in operational taxonomic unit (OTU) density within the soil samples had been sufficiently captured at the sequencing depth used (Fig. S1). Therefore, the data were suitable for analysis of the microbial communities. In total, 2,053,554 bacterial 16S rRNA gene reads were obtained from 18 sequencing samples with an average length of 433 bp. OTUs are the taxa of particular classification groups based on the similarity between nucleotide sequences. We classified 3,948 bacterial OTUs (Table 2), which were clustered into 31 bacterial phyla. Fungal 18S rRNA yielded 326 fungal OTUs that were clustered into 21 phyla.

**Table 2:** Number of sequences analysed, observed diversity richness and diversity/richness indices of the 16S rRNA bacterial and 18S rRNA fungal libraries obtained for clustering at 97% identity.

Sample	Bacteria			Fungi		
	OTUs	chao1	Shannon	OTUs	chao1	Shannon
KE	1784 ± 58ab	2227 ± 92ab	5.79 ± 0.06b	147 ± 9.64bc	169 ± 7.93ab	3.27 ± 0.11b
NE	1795 ± 136ab	2214 ± 102ab	5.82 ± 0.11b	136 ± 5.03bc	159 ± 7.388b	3 ± 0.15c
KP	1700 ± 70bc	2131 ± 90ab	5.77 ± 0.14b	158 ± 1.53ab	176 ± 2.52ab	3.16 ± 0.05bc
NP	1615 ± 44c	1987 ± 35c	5.75 ± 0.11b	129 ± 9.85c	156 ± 25.64b	3 ± 0.09c
KS	1915 ± 39a	2321 ± 35a	6.12 ± 0.01a	178 ± 8.66a	204 ± 2.61a	3.22 ± 0.08bc
NS	1928 ± 130a	2322 ± 142a	5.87 ± 0.17ab	153 ± 29.70b	172 ± 47.95ab	3.44 ± 0.01a

KE: *E. robusta* in karst areas; NE: *E. robusta* in non karst areas; KP: *P. yunnanensis* in karst areas; NP: *P. yunnanensis* in non karst areas; KS: secondary forest in karst areas; NS: secondary forest in non karst areas. Data is reported as mean ± standard error (n = 3); Means with different letters within a column are significantly different at *P* < 0.05.

The Shannon and Chao1 indices were used to represent species diversity and richness estimates. As shown in Table 2, the OTU numbers and both indices in the microbial community were statistically identical between the karst and non-karst ecosystems within the same forest type. The Chao1 index of KP was slightly higher than its non-karst counterpart. The numbers of bacterial and fungal OTUs were 1,615–1,928 and 129–178, respectively. The lowest numbers of bacterial and fungal OTUs were observed in NP. The Shannon index in bacteria was higher (6.12) in KS than in other treatments. Additionally, the Shannon index in fungi in NS was 3.44, which was higher than that in NP.

### Taxonomic distributions of identified bacteria and fungi

**Figure 1:**
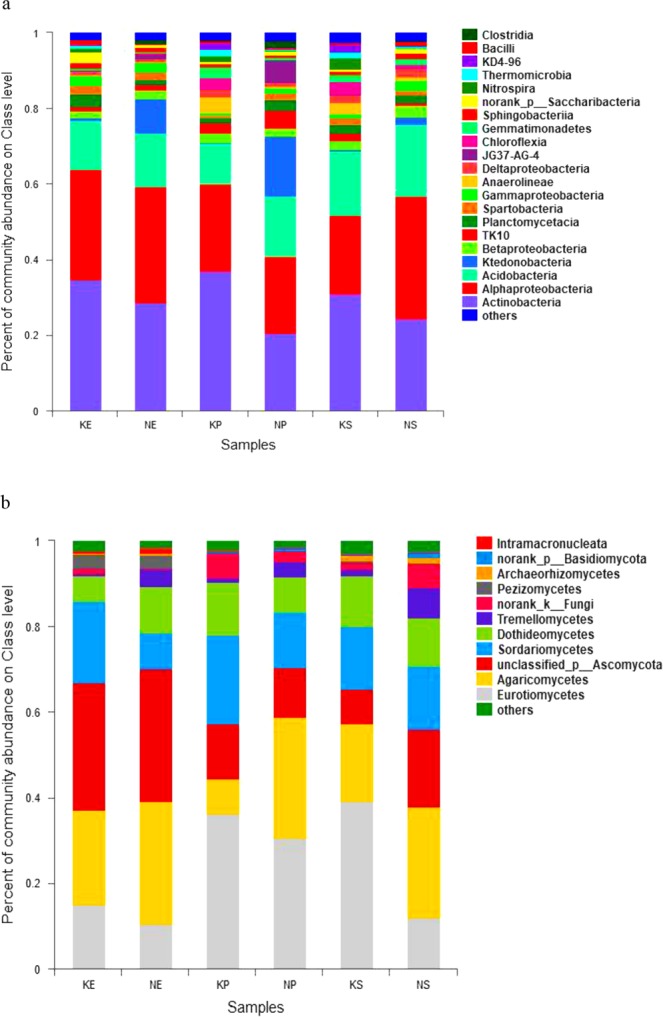
Relative abundances of the dominant bacterial (**a**) and fungal (**b**) groups in each sample at class level. KE: *E. robusta* in karst areas; NE: *E. robusta* in non karst areas; KP: *P. yunnanensis* in karst areas; NP: *P. yunnanensis* in non karst areas; KS: secondary forest in karst areas; NS: secondary forest in non karst areas.

We analyzed the OTUs to determine the taxonomic distribution of the identified bacteria at the genus, phylum, and class levels. A comparison of the relative abundances of rhizosphere resident microbial genera indicated that they were influenced by the geological conditions and vegetation type (Fig. S2). However, we were unable to identify most of the microbes at the genus level. At the phylum level, the bacterial community composition was similar among the different plots, with Proteobacteria, Actinobacteria, Acidobacteria, and Chloroflexia being the most abundant phyla in the soils in all plots (Fig. S3a). While the distribution of each class varied, all sequences could be classified into 22 groups (those with abundances <1% were classified into ‘others’). The five most dominant bacterial classes among all soils were Actinobacteria, Alphaproteobacteria, Acidobacteria, Ktedonobacteria, and Betaproteobacteria, accounting for >70% of the total reads (Fig. 1a).

Ascomycota and Basidiomycota dominated the fungal community at the phylum level in all of the soils (Fig. S3b). A total of 21 fungal classes were observed in the soil samples, which were largely distributed among the classes of Eurotiomycetes, Agaricomycetes, Sordariomycetes, Dothideomycetes, and ‘unknown in the phylum Ascomycota’ (total >80%). To investigate the differences of fungal and bacterial OTUs under different settings, we used heatmap analysis of the 20 most abundant OTUs, which highlighted their relative distributions and abundances (Fig. S4). The relative abundances of these 20 dominant OTUs differed among the six samples and in each vegetation type. Influenced by geological background conditions, karst lands had more OTU2495_b, OTU1704_b, and OTU2655_b in bacteria and more OTU224_f and OTU252_f in fungi. This observation was in agreement with the finding of Paterson *et al*.^25^ that vegetation type had a profound effect on soil communities and processes, particularly those in the rhizosphere. Additionally, the significant differences in microorganism structure between karst and non-karst ecosystems were consistent with the findings of a previous study using similar experimental approaches^26^.

### Community structure and Bray-Curtis dissimilarity

**Figure 2:**
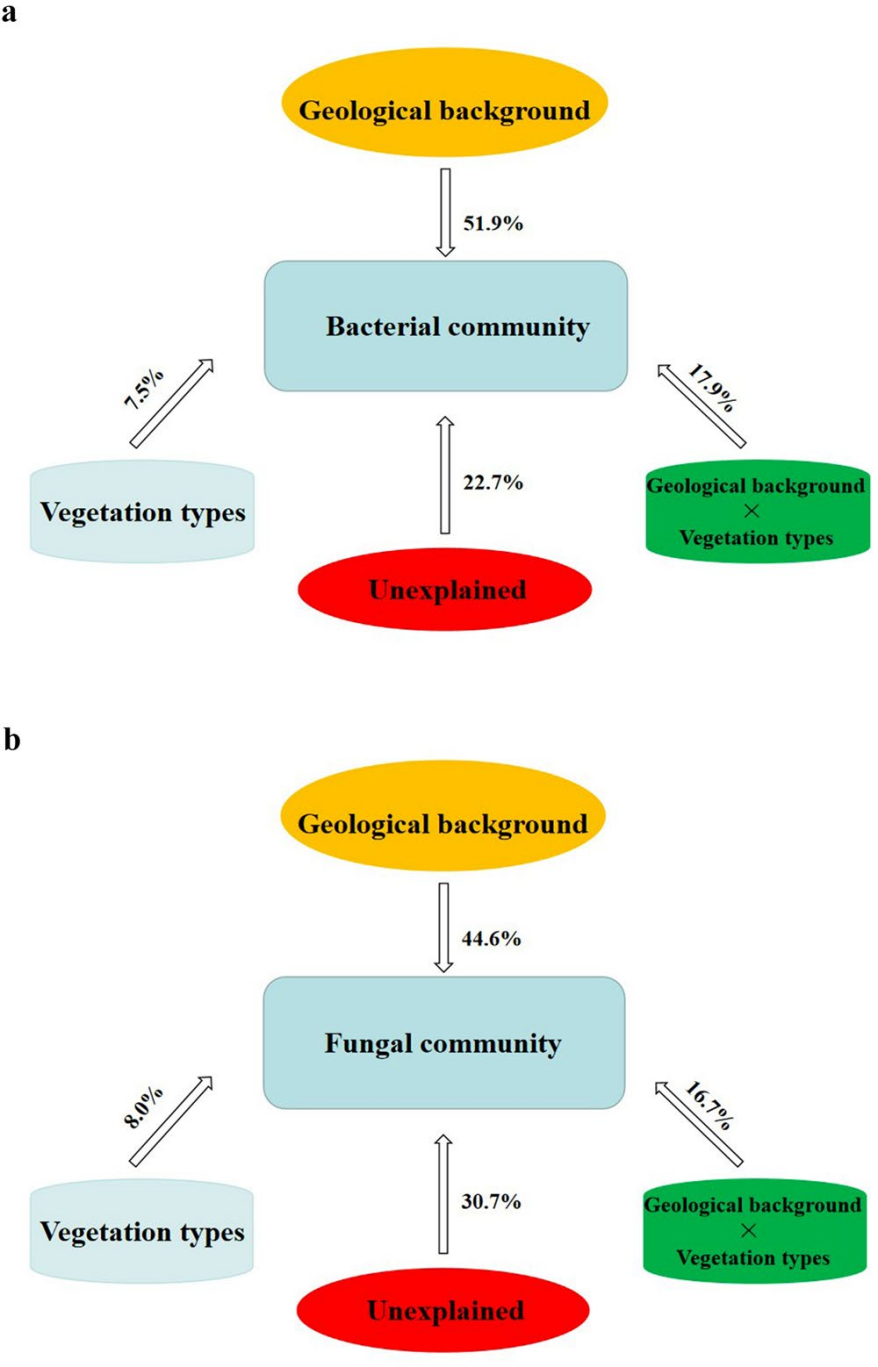
Variation partitioning analysis (VPA) differentiating effects of microbial community ((**a**) bacteria; (**b**) fungi).

From Fig. 2, we found that the independent effects of two factors (vegetation type and geological background) and their interaction on the microbial community were different. In bacteria, a total of 77.3% of the variation could be explained by the selected variables. Geological background explained 51.9% of the variation, vegetation type explained 7.5%, and the interaction between geological background and vegetation type explained 17.9%. In fungi, 69.3% of the variation could be explained by the selected variables. Geological background explained 44.6%, vegetation type explained 8.0%, and the interaction between geological background and vegetation type explained 16.7%.

**Figure 3:**
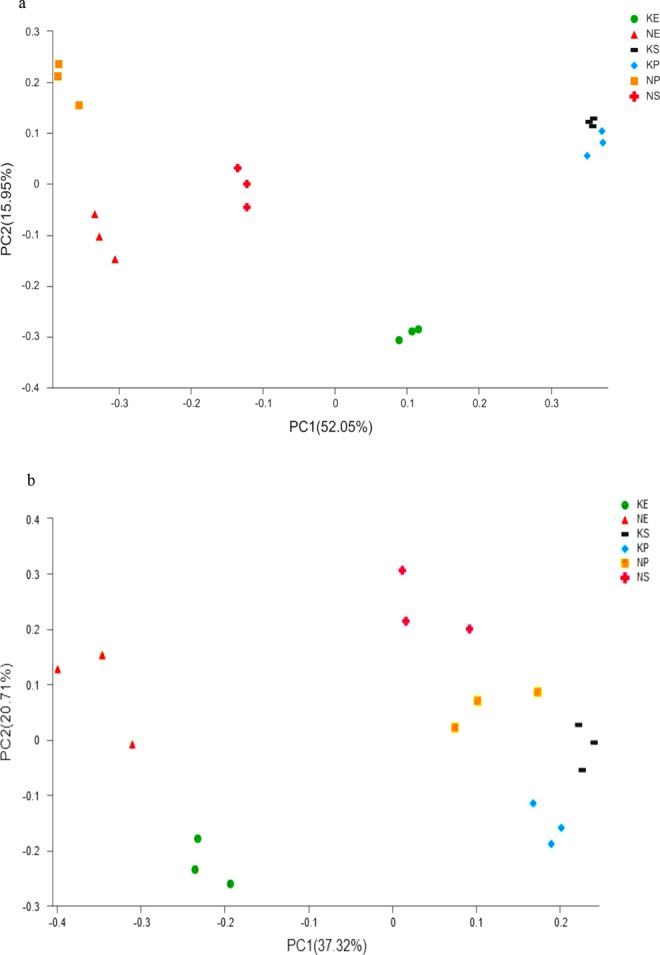
Principal coordinate analysis of microbial community composition in soil ((**a**) bacteria; (**b**) fungi). KE: *E. robusta* in karst areas; NE: *E. robusta* in non karst areas; KP: *P. yunnanensis* in karst areas; NP: *P. yunnanensis* in non karst areas; KS: secondary forest in karst areas; NS: secondary forest in non karst areas.

**Figure 4:**
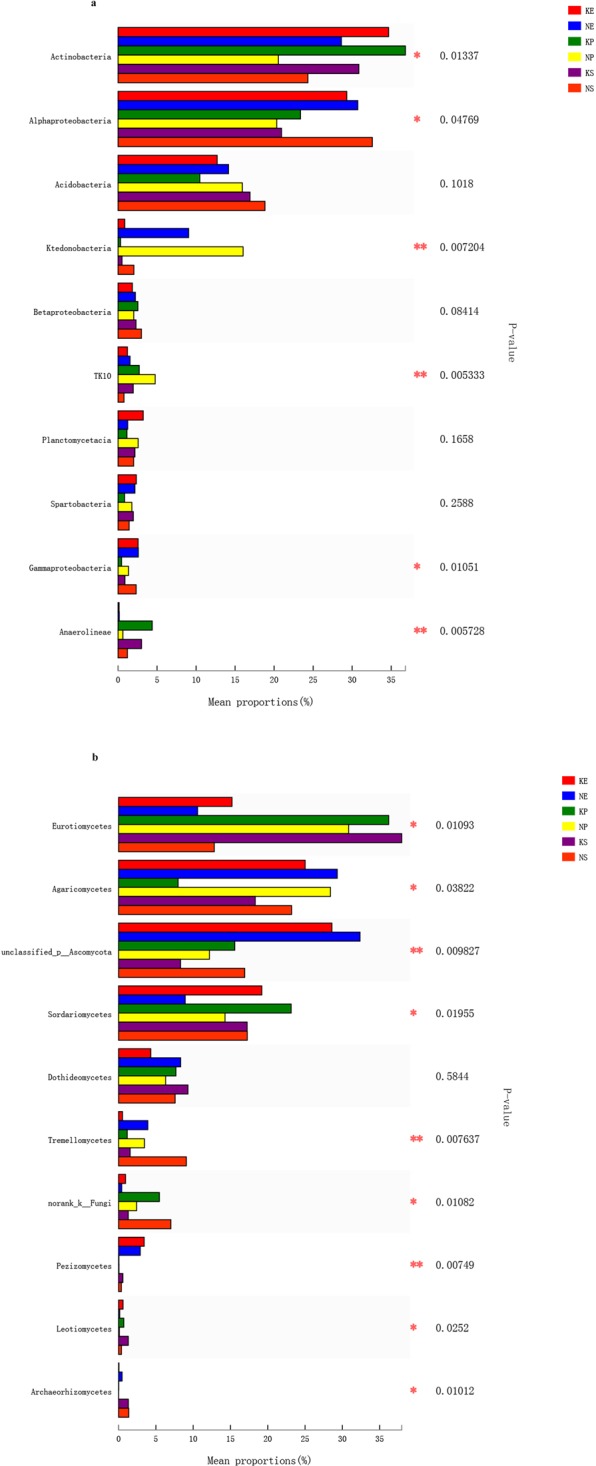
The differences of the relative abundances of microbial communities at a class level among six samples ((**a**) bacteria; (**b**) fungi). KE: *E. robusta* in karst areas; NE: *E. robusta* in non karst areas; KP: *P. yunnanensis* in karst areas; NP: *P. yunnanensis* in non karst areas; KS: secondary forest in karst areas; NS: secondary forest in non karst areas. ^***^P < 0.001; ^**^P < 0.01; ^*^P < 0.05.

To examine differences in microbial communities among the soil samples further, we calculated the dissimilarity in the community composition at the OTU level. PCoA based on Bray-Curtis dissimilarity showed that bacterial and fungal communities significantly differed among sites (Fig. 3). The two principal coordinates (PCs) explained 67% of the total variance in the bacterial community (Fig. 3a), with PC1 and PC2 explaining 52.05% and 15.95% of the total variance, respectively. The average Bray-Curtis dissimilarity of all fungal communities in the six plots was 58.03% (Fig. 3b). Compared with the non-karst ecosystems, the soil microbial community composition and structure in the rhizosphere of *P. yunnanensis* were more similar to those in the natural forest than to the *E. robusta* plantation in the karst ecosystems. There were significant differences in six bacterial (Actinobacteria, Alphaproteobacteria, Ktedonobacteria, TK10, Gammaproteobacteria, and Anaerolineae) and nine fungal (Eurotiomycetes, Agaricomycetes, unclassified _p_Ascomycota, Sordariomycetes, Tremellomycetes, norank_k_Fungi, Pezizomycetes, Leotiomycetes and Archaeorhizomycetes) classes among the soils collected from the six plots. With the same kind plant species, more Actinobacteria (30.93–36.71%) and fewer Ktedonobacteria, were detected in the karst soils than in the non-karst samples (Fig. 4a). The dominant bacterial species (i.e., the relative abundances of Actinobacteria, Alphaproteobacteria, and Anaerolineae) were also affected by the forest type in the same background. Additionally, most fungal classes were also influenced by the vegetation type and geological background condition (Fig. 4b).

Two-way analysis of variance (ANOVA) was used to study the effect of the interaction between geological condition and vegetation type on the bacterial (three most abundant) and fungal (five most abundant) community compositions in the soil samples. Actinobacteria, Alphaproteobacteria, Sordariomycetes, Eurotiomycetes, and Dothideomycetes were significantly affected by the interaction between geological condition and vegetation type (Table 3).

**Table 3:** Effect of the geological type and vegetation type on bacteria and fungi.

	Bacteria				Fungi			
	Actinobacteria	Alphaproteobacteria	Acidobacteria	Eurotionmycetes	Agaricomycetes	Sordariomycetes	Dothideomycetes	unclassified-Ascomycota
Geological type × Vegetation type	^*^	^*^	NS	^***^	NS	^*^	^*^	^*^

^***^P < 0.001; ^**^P < 0.01; ^*^P < 0.05; NS, not significant.

**Figure 5:**
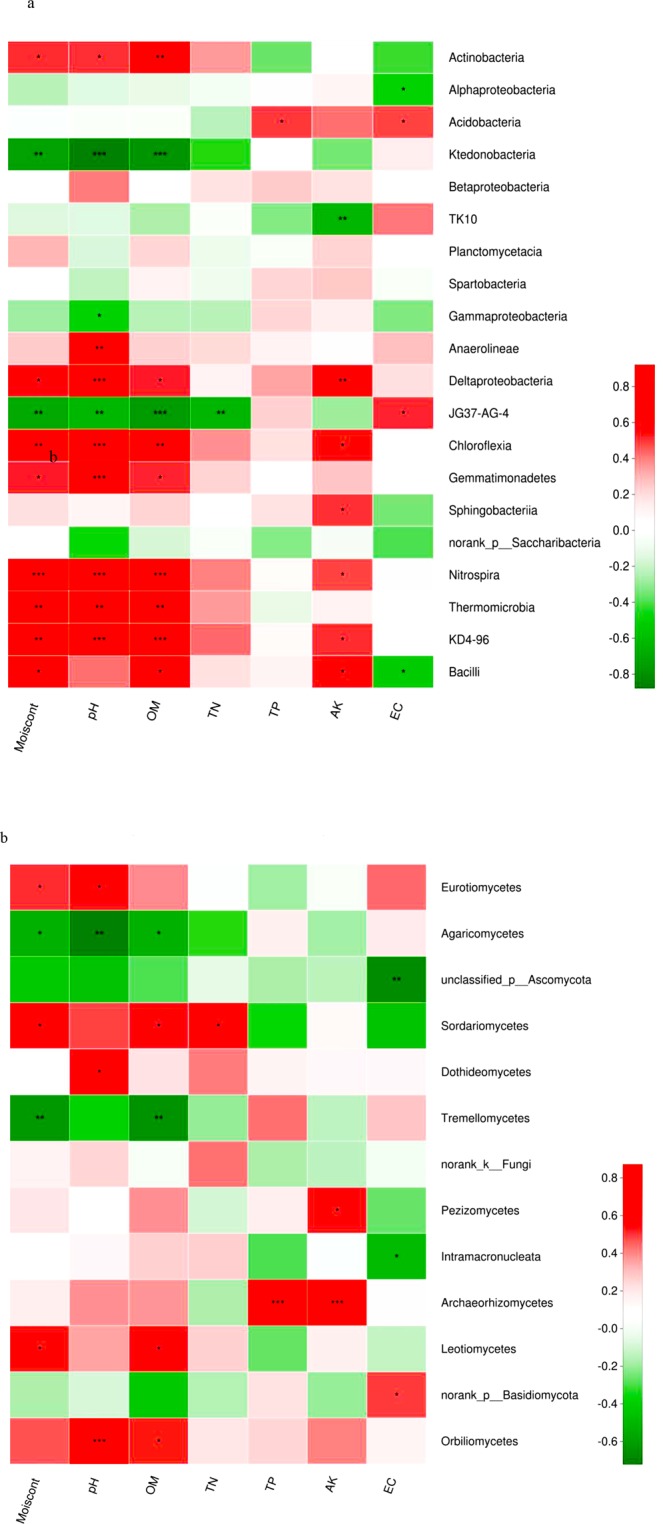
Relationships among the relative abundance of microbial communities and physicochemical parameters based on the Spearman correlation analysis ((**a**) bacteria; (**b**) fungi). AK: available potassium; TN: total nitrogen; TP: total phosphorus; OM: organic matter; EC: electrical conductivity. ^***^P < 0.001; ^**^P < 0.01; ^*^P < 0.05.

A Spearman correlation heatmap was used to examine the effects of soil factors on bacterial and fungal composition at the class level. The correlation between microorganism composition and all measured parameters showed a clear gradient, as shown in Fig. 5. From Fig. 5a, it can be seen that OM, pH and moisture content were significantly correlated with several of the bacterial communities (the top 20 at the class level). The relative abundances of Thermomicrobia, Nitrospira, Chloroflexia, Deltaproteobacteria, Anaerolineae, Actinobacteria, Gemmatimonadetes, Gammaproteobacteria, and Ktedonobacteria were significantly affected by pH, and the latter two classes displayed a negative correlation with pH. The OM content displayed a significant positive correlation with the relative abundances of Bacilli, Thermomicrobia, Nitrospira, Chloroflexia, Gemmatimonadetes, Deltaproteobacteria, and Actinobacteria, but it was negatively correlated with the abundance of Ktedonobacteria. In addition, the relative abundances of Bacilli, Thermomicrobia, Nitrospira, Chloroflexia, Gemmatimonadetes, Deltaproteobacteria, and Actinobacteria were inversely proportional to the water content of the soils.

The OM content, pH, and moisture content were significantly correlated with most of the top 13 classes of fungi (Fig. 5b). Spearman correlation analysis revealed that Orbiliomycetes were significantly correlated with pH and OM, and that Archaeorhizomycetes were significantly correlated with TP and AK. Tremellomycetes were negatively correlated with OM and water content. The relative abundance of Agaricomycetes was influenced by OM, pH, and water content, and Eurotiomycetes displayed a significant positive correlation with pH and water content.

## Discussion

Karst ecosystems are threatened by rapid degradation processes compared to non-karst ecosystems, such as soil loss and reduced water holding capacity, resulting in changes in vegetation cover that can be difficult to reverse^27,28^. Changes in soil microbial community structure are an important indicator of the health and sustainability of an ecosystem and are also used as an indicator of soil quality in many ecosystem processes^29–31^. Therefore, understanding shifts in soil microbial community structure under complex environmental conditions is vital for effective vegetation restoration in karst areas^32^. In this study, we used molecular techniques to evaluate the effects of geological conditions and vegetation type on soil microbial structure and composition.

### Effects of geological background conditions on microbial community structure and diversity

We used diversity and richness indices to examine variability in microbial communities in different rhizosphere soils between karst and non-karst areas. From Table 2, it can be seen that the indices (OTU number, Shannon and Chao1) generally show no significant difference in bacterial or fungal community between the karst and non-karst areas. This may be due to the tolerance for natural disturbance, and self-recovery and regeneration processes, despite the fact that karst forests are a relatively fragile ecosystem type^33^.

The interactions between carbonate rocks and microbes can increase dissolution and release calcium ions (Ca^2+^), and enhance the content of trace elements in the soil matrix, which helps to maintain soil microbial growth and stimulate the secretion of extracellular enzymes^3,34,35^. Liang *et al*.^29^ demonstrated that bacterial richness and diversity had a significantly positive correlation with the available Ca^2+^ content, which implied that the Ca^2+^ content determined soil bacterial populations and activity. In our study, the soil microbial community composition differed between the karst and non-karst areas (Fig. 3). In the bacteria, the relative abundances of Actinobacteria, Chloroflexia, and Gemmatimonadetes were higher in the karst ecosystems, and the percentages of Ktedonobacteria were higher in the non-karst ecosystems (Figs 1a and 4a). Ktedonobacteria were negatively correlated with OM content, and the karst areas had higher OM contents (Table 2, Fig. 5a). In the karst areas, vegetation litter and plant residues were less impacted, and microbial decomposition of these more abundant residues led to the higher OM content in the karst lands. Yuan *et al*.^35^ reported that Actinobacteria was dominant in soil samples collected from weathered rock areas. Actinobacteria are commonly found in cave environments, such as the weathered rocks of the Buda Thermal Karst System^36^. The abundances of Gemmatimonadetes were significantly higher in karst than in non-karst ecosystems. DeBruyn *et al*.^37^ demonstrated that the relative abundances of Gemmatimonadetes were related to their adaptation to a dry environment. Thus, their higher relative abundances in karst areas may be due to the poor water-retention ability of soils in karst ecosystems. Chloroflexia were also identified in our study; these were originally thought to only inhabit extreme environments, but were recently found in the endolithic communities of dolomite and limestone rocks^38^.

In the fungi, we observed higher relative abundances of phylum Ascomycota, including Eurotiomycetes, Sordariomycetes and Leotiomycetes, in the karst soils (Figs 1b and 4b). Tang and Lian^39^ showed that Ascomycota were generally dominant in rocky habitats, indicating that their presence may be beneficial to the weathering of the rock. Interestingly, members of the phylum Basidiomycota, such as Agaricomycetes and Tremellomycetes, were more abundant in the non-karst soils. Both of these phyla were dominant in all of the samples (Fig. S3b), and their dominance has been associated with cellulose degradation in agricultural soils^40^ via facilitating the use of the soil’s various lignin components by plants. However, the proportions of Ascomycota and Basidiomycota communities in the soils displayed low similarity between the karst and non-karst areas, because each species has its own suitable physical and chemical conditions and their abundance is restricted by the growth environment^41^.

### Effects of recovery patterns on microbial community structure and diversity

Microbes play an essential role in ecosystem recovery, and the soil microbial community composition is in turn affected by the plants growing in the soil (which provide resources for the microbial community)^31,42^. Thomson^43^ suggested that different plants recover at different rates and grow in different directions, leading to changes in the relative abundances of certain taxa. In this study, bacterial and fungal classes displayed differences among the three vegetation treatments in the same background (Figs 3 and 4). The variation in microbial communities may be due to the differences in root system architecture and exudates of the different plant species, which determined the differential selection of microbial partners from the soil environment^11,44^. Li *et al*.^45^ reported that indigenous trees restored soil microbial biomass at faster rates than exotic species. In our study, in the karst ecosystems, the soil microbial community composition and structure in the rhizosphere of *P. yunnanensis* were more similar to those of the natural secondary forests (Fig. 3).This may be due to direct competition between the introduced exotic species and native species, which reduces the biodiversity and thus creates a less favorable condition for native plant species^46^. However, the composition of the microbial community structure in the *E. robusta* artificial forests was markedly different from that in the natural secondary forest. Another possible factor influencing the microbial community in the *P. yunnanensis* forest is the fact that *P. yunnanensis* was the dominant tree species growing naturally in this area and adapts more easily to the local natural environment with less interference in biodiversity for the native microbial community^47^, even when it is growing in plantation forest.

In karst ecosystems, fungi and bacteria interact to promote the weathering of rocks, which accelerates soil formation^2^. In this context, our results showed that the rhizosphere bacterial community structure mainly comprised Proteobacteria, Actinobacteria, Acidobacteria, and Chloroflexia at the phylum level (Fig. S3a). Proteobacteria and Actinobacteria are known copiotrophs, which have high nutritional requirements^12^, and the members of Acidobacteria exhibit oligotrophic attributes^48^. The fungal community contained the Ascomycota and Basidiomycota phyla (Fig. S3b). The dominant microbial communities at the phylum level in the karst soils from the six treatments in our study were similar to those found in other studies in karst soils^2,35^. In karst lands, fungi play important roles in many essential processes, including OM decomposition, element release by mineralization, and protection against leaching by element storage in biomass^49^. Lian *et al*.^2^ described how fungal mycelia inserted into rock crevices absorbed moisture from microcracks in the rock, which kept the borehole in the surface of the carbonate rock relatively moist; thus, acid secretions from the bacteria could dissolve fully within the rock, accelerating its breakdown.

### Effects of physicochemical factors on microbial community structure

Microbial physiology and community structure are affected by environmental conditions^31,50^. In our study, soil pH and OM content played a critical role in shaping the microbial community composition (Fig. 5), consistent with the findings of Osborne *et al*.^44^ and Smith *et al*.^19^. Furthermore, Fierer and Jackson^51^ reported that pH influenced the primarily soil microbial communities in karst ecosystem revegetation sites. Additionally, our study demonstrated that the distribution of Actinobacteria was significantly affected by soil pH, in agreement with Tripathi *et al*.^52^. The strong correlation between soil pH and microbial community structure may be explained by the relatively narrow growth tolerances exhibited by most microorganisms and the harsh growing conditions in the karst environments. OM supports microbial growth and reproduction, and most microorganisms showed a significant positive correlation with OM content; however, a few microbe classes were negatively correlated with OM content, including Ktedonobacteria, Agaricomycetes and Tremellomycetes. This may be due to reduced competitiveness among these microbes under the nutrient-rich conditions.

Restoration of vegetation can enhance soil organic carbon and nitrogen dynamics by increasing soil OM input and the water-holding capacity of the soil. Liang *et al*.^30^ reported that vegetation-associated soil properties played a vital role in determining bacterial community composition. Our findings indicate that *P. yunnanensis*, which is indigenous to the study area, was capable of restoring soil health in terms of the desired microbial diversity and yielded soil similar to that in the natural secondary forest. This suggests that planting *P. yunnanensis* in natural secondary forest promotes rapid restoration of the ecological environment by reducing disturbance of the soil microbial community.

## Conclusions

Our results indicated that the microbial diversity generally showed no significant difference in bacterial and fungal communities between the karst and non-karst areas. In the karst areas, the *P. yunnanensis* forest plantation displayed a microbial community structure that was more similar to the natural secondary forest than to the *E. robusta* plantation, indicating that the former species may be a more effective choice for ecological restoration. Our study highlights the importance of considering the relationships among geological conditions, vegetation, and microbiota in the environmental management of karst areas. However, the specific functions of the microbes distributed in different environments remain unclear. Further studies are required to examine the functional differences among different microbial communities using metagenome sequencing, which would provide further information to guide effective environmental management in vulnerable karst regions. The findings of our study will provide important information that can guide appropriate management and vegetation restoration strategies for degraded karst ecosystems. We expect to combine functional microorganism and vegetation restoration strategies to guide appropriate management in degraded karst ecosystems, promoting the weathering of rocks and accelerating soil formation.

## Materials and Methods

### Site description and sampling

The study site is located within the Jianshui karst ecosystem of the National Field Research Station, Key Laboratory of the State Forestry Administration for Soil and Water Conservation, Yunnan Province, China (102°54′12″E, 23°37′13″N). The site is characterized by a subtropical monsoon climate. The mean annual precipitation is 805 mm, which is mainly distributed from May to October. The Jianshui karst tectonic basin contains two main geological environments: karst and non-karst areas. The total area of the basin is 3,940 km^2^, 68.77% of which is covered by karst. Due to their characteristics, the carbonate rocks of the karst landscapes provide a significant survival challenge for most organisms. Based on the geological conditions, we selected three representative vegetation types, two areas of artificial forest and one of naturally regenerated secondary forest, which were all found in both the karst and non-karst areas: two areas of artificial forest, and one of naturally regenerated secondary forest. The two areas of artificial forest contained *Eucalyptus robusta* (*E. robusta*) and *Pinus yunnanensis* (*P. yunnanensis*), respectively. *E. robusta* is a fast-growing woody plant widely used for reforestation worldwide. *P. yunnanensis* is a native species in Yunnan, and is tolerant of soils with low fertility. Since 1996, *E. robusta* and *P. yunnanensis* plantations have been planted at large-scale in this region. The natural secondary forest was dominated by *Quercus* species.

Three replicate plots were selected for each vegetation type, total 18 research plots (2 geological conditions × 3 plant species × 3 replicates; KE: *E. robusta* in karst area; NE: *E. robusta* in non-karst area; KP: *P. yunnanensis* in karst area; NP: *P. yunnanensis* in non-karst area; KS: secondary forest in karst area; and NS: secondary forest in non-karst area). A 20 m × 20 m horizontal projection area^30^ was set up for each plot in June 2016. All sites were located at least 1,000 m from the forest edge. Rhizosphere soil samples were collected in May 2017 from the rhizosphere of plants in the plots. However, the growth of vegetation affects the physical and chemical properties of soil and the microbial communities. Thus, to maintain the homogeneity of soil samples, all selected trees in each plot were similar (i.e., same age and similar diameter)^53^. After removing the surface litterfall, the soil adhering to plant roots (*ca* 1–2 mm on root) was collected from different locations within each plot, and five samples were systematically pooled together into one composite sample. A total of 18 mixed soil samples were collected for the purpose of the study. A subsample from each of these mixed soil samples was stored at 4 °C and its physical and chemical properties were analyzed. The remaining portion of each sample was stored at −80 °C for analysis of the microbial community structure.

### Analysis of soil physicochemical properties

Soil moisture content was determined using a gravimetric method after drying the fresh soil in an oven at 105 °C for 24 h, pH value and electrical conductivity (EC) were measured using a 1:10 (w/v) soil-water slurry with a pH meter (PB-10; Sartorius, Göttingen, Germany) and an EC meter (DDS-307A; Rex Shanghai, Shanghai, China), respectively. The soil OM and total nitrogen (TN) contents were measured using dichromate oxidation and the Kjeldahl method, respectively. Available potassium (AK) was determined using flame photometry^54^. Total phosphorus (TP) was analyzed colorimetrically using the ammonium molybdate method^55^.

### Microbial diversity analysis

For each soil sample, microbial DNA was extracted from 0.2 g of soil using a fast DNA® spin kit (Omega Bio-Tek, Norcross, GA, USA) according to the manufacturer’s directions and was checked using 1% agarose gel electrophoresis and spectrophotometry (three replicates for each sample). The V3-V4 region of the bacteria 16S rRNA gene was amplified using polymerase chain reaction (PCR) analysis (95 °C for 2 min, followed by 25 cycles at 95 °C for 30 s, 55 °C for 30 s, and 72 °C for 30 s, with a final extension of 72 °C for 5 min) using the primers 338 F 5′-barcode- ACTCCTACGGGAGGCAGCAG-3′ and 806 R 5′-GGACTACHVGGGTWTCTAAT-3′^56^. Additionally, the fungal 18S rRNA gene was amplified using the primers SSU0817F 5′-barcode-TTAGCATGGAATAATRRAATAGGA-3′ and 1196 R 5′-TCTGGACCTGGTGAGTTTCC-3′^57^. The PCR reactions were performed in triplicate in a 20 μL mixture, containing 4 μL of 5 × FastPfu Buffer, 2 μL of 2.5 mM dNTPs, 0.8 μL of each primer (5 μM), 0.4 μL of FastPfu Polymerase, and 10 ng of template DNA.

Amplicons were extracted from 2% agarose gel and purified using an AxyPrep DNA Gel Extraction Kit (Axygen Biosciences, Union City, CA, USA), and quantified using QuantiFluor™-ST (Promega, Madison, WI, USA). The purified amplicons were pooled together in equimolar quantities and paired-end sequenced (2 × 250) on an Illumina MiSeq platform following the standard protocols before sequencing.

Then, we analyzed high-quality sequences for which the raw sequences were first demultiplexed and quality-filtered using QIIME (ver. 1.17), based on the sequence length, quality, primers, and tags. The unique sequence set was classified into operational taxonomic units (OTUs) with 97% similarity using UPARSE (ver. 7.1 http://drive5.com/uparse/), and the chimeric sequences were identified and removed with UCHIME. To estimate alpha diversity, the sequence of all samples was rarefied to the minimum sequences and subsequent statistical analyses were based on the rarefied data. The taxonomy of each 16S rRNA gene sequence was analyzed using the RDP Classifier (http://rdp.cme.msu.edu/) against the SILVA (SSU115)16S rRNA database using a 70% confidence threshold^58^. For fungi, the phylogenetic assignment was determined against the UNITE database release 5.0^16^ and the NCBI. All sequences were deposited into the Sequence Read Archive (SRA) under the accession numbers. SRP180206 for bacteria and SRP180210 for fungi.

### Statistical analysis

Data were arranged in spreadsheets in Excel 2007 (Microsoft Corp., Redmond, WA, USA). The significance of differences in soil properties and alpha diversity was tested using one-way analysis of variance (ANOVA) followed by a least significant difference (LSD) test at 95% confidence level. Variation partitioning analysis (VPA) was performed using Canoco5 software. All high throughput DNA sequencing data were analyzed on the Majorbio I-Sanger Cloud Platform (http://www.i-sanger.com/).

## Supplementary information

Supplementary information
