# Supplementary material for: Changes in Plant Rhizosphere Microbial Communities under Different Vegetation Restoration Patterns in Karst and Non-karst Ecosystems
Source: Sci Rep. 2019 Jun 19;9:8761. doi: 10.1038/s41598-019-44985-8 (PMC6584648; doi:10.1038/s41598-019-44985-8)
Supplement: Supplementary file 1 — Supplementary information [file 41598_2019_44985_MOESM1_ESM.doc]

Supplementary information

**Changes in Plant Rhizosphere Microbial Communities under Different Vegetation Restoration Patterns in Karst and Non-karst Ecosystems**

Zhouzhou Fan^1^, Shuyu Lu^1^, Shuang Liu^1^, Hui Guo^1^, Tao Wang^2^, Jinxing Zhou^3^ & Xiawei Peng^1^


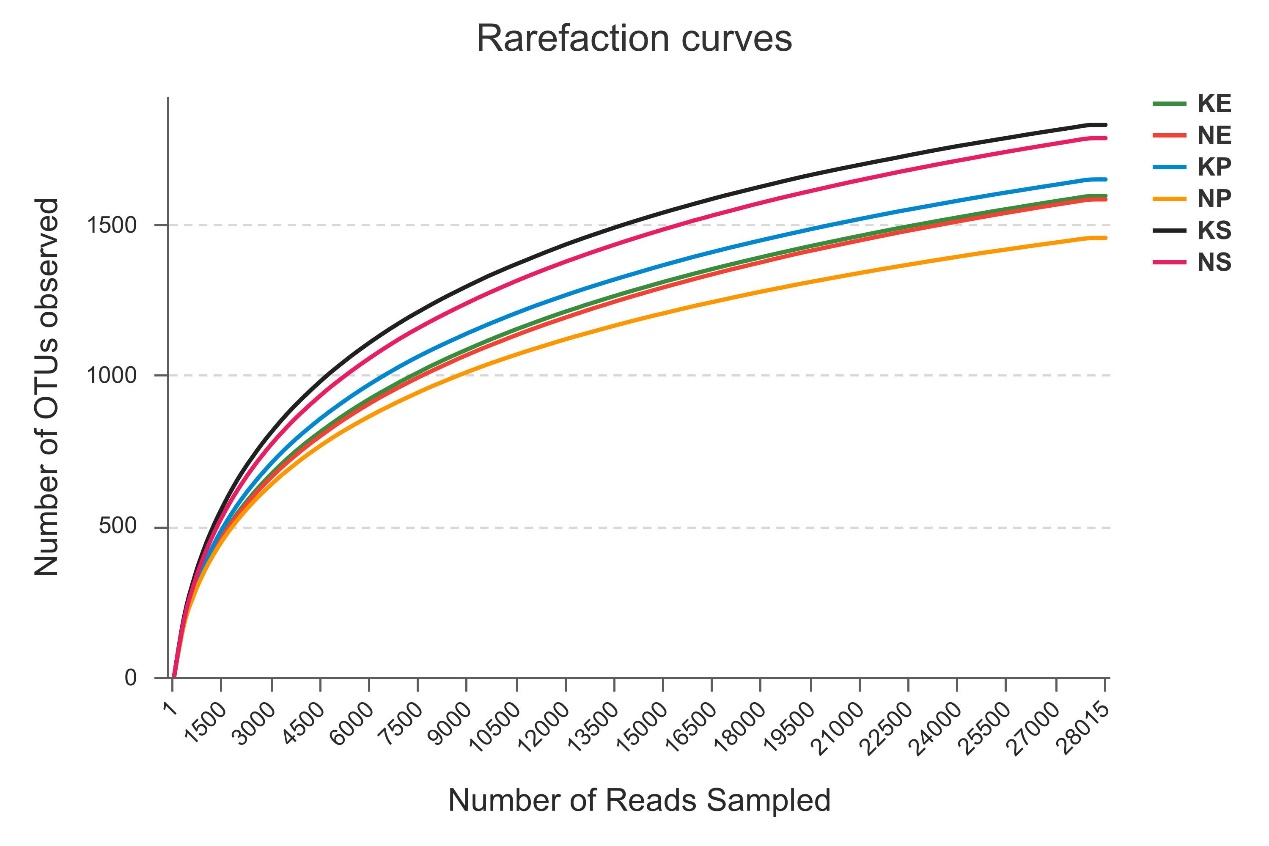


a


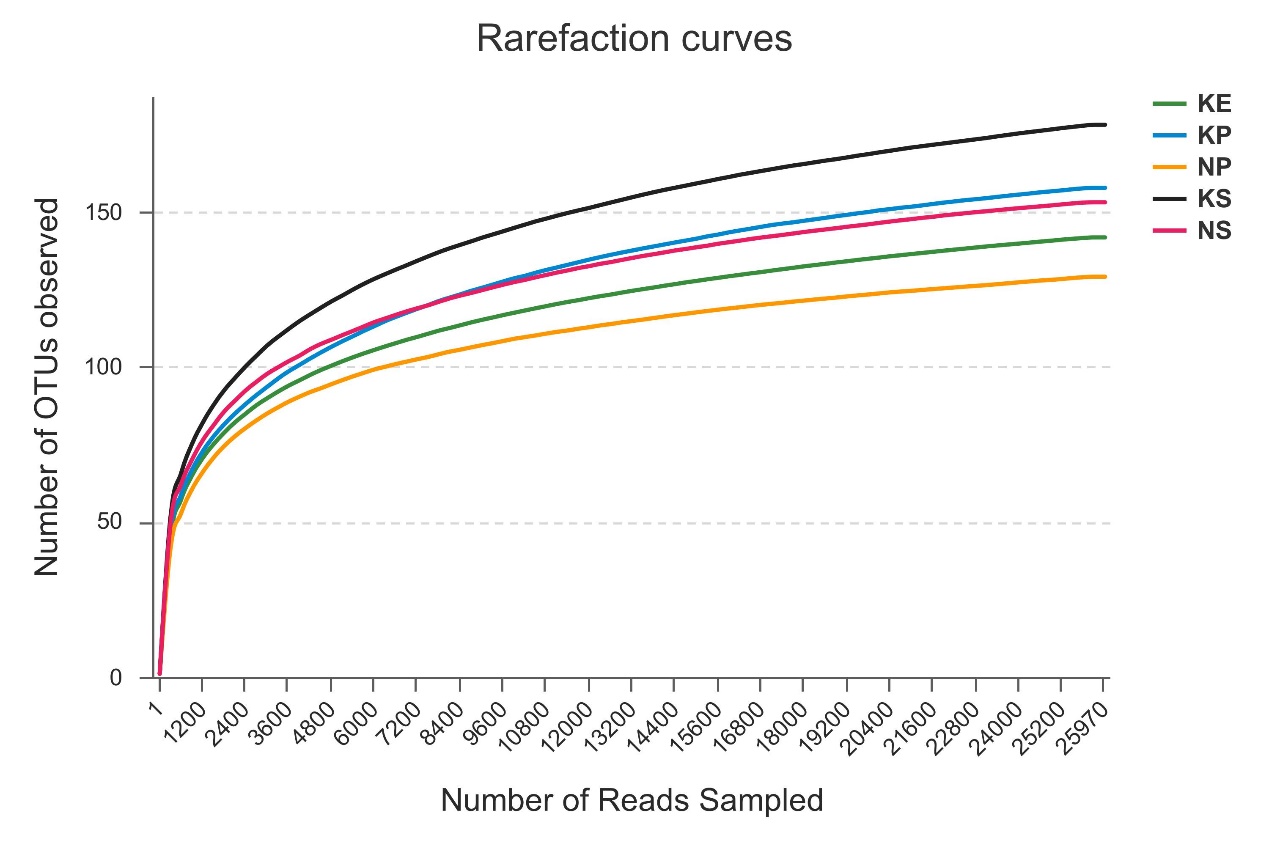


b

**Fig. S1.** Rarefaction curves of bacterial and fungal communities in tested soils at a 3% distance cut-off (a: bacteria; b: fungi).

a


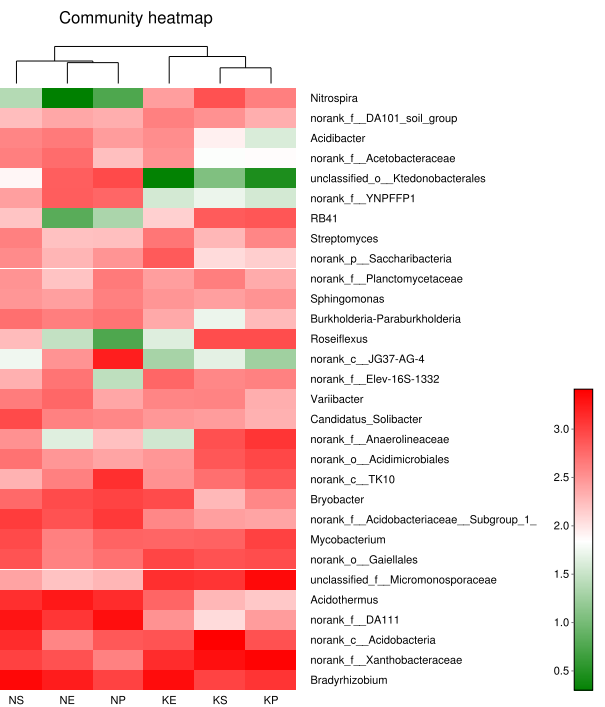


b


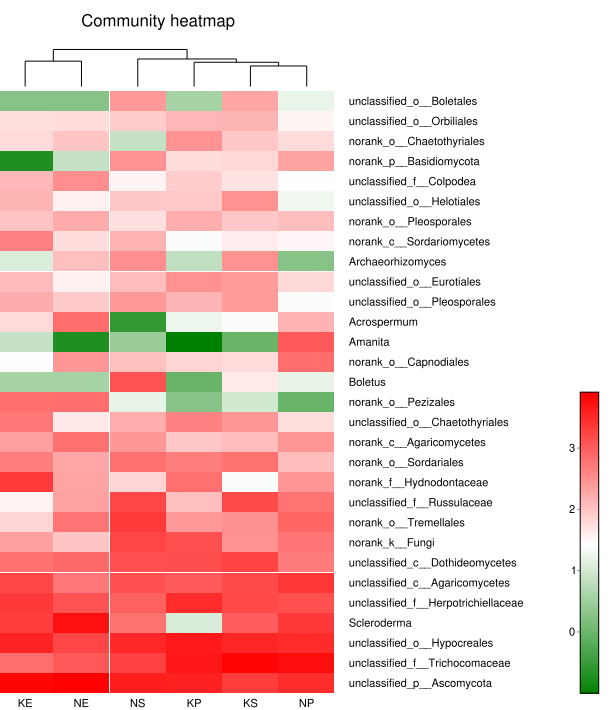


**Fig. S2.** Community heatmap showing top 30 microbial genus in all sample (a: bacteria; b: fungi).


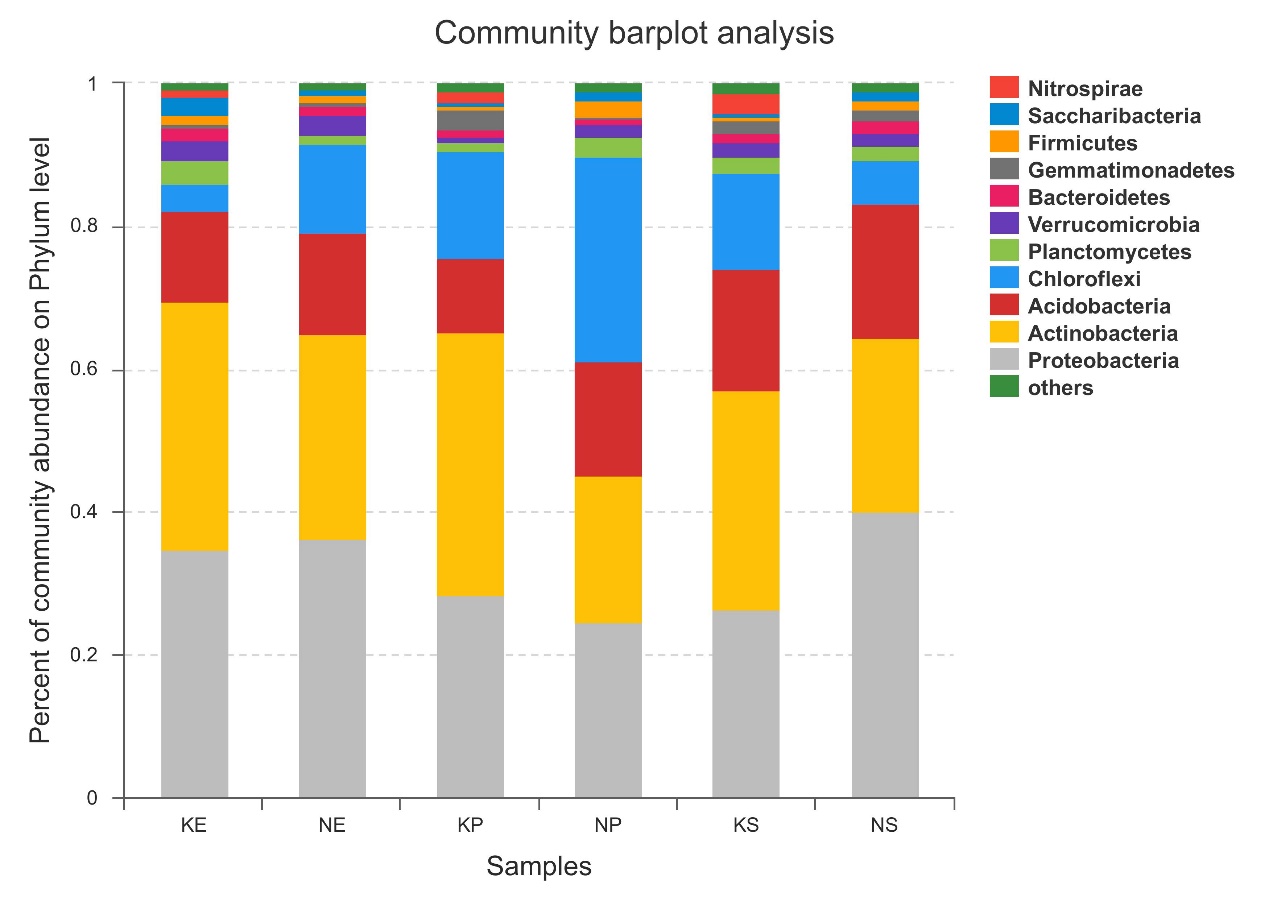


b

a


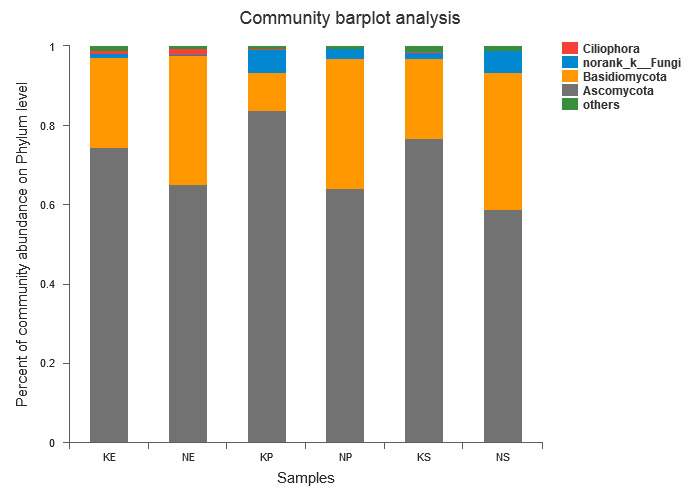


**Fig. S3.** Relative abundances of the dominant bacterial (a) and fungal (b) groups in each soil at phylum level.


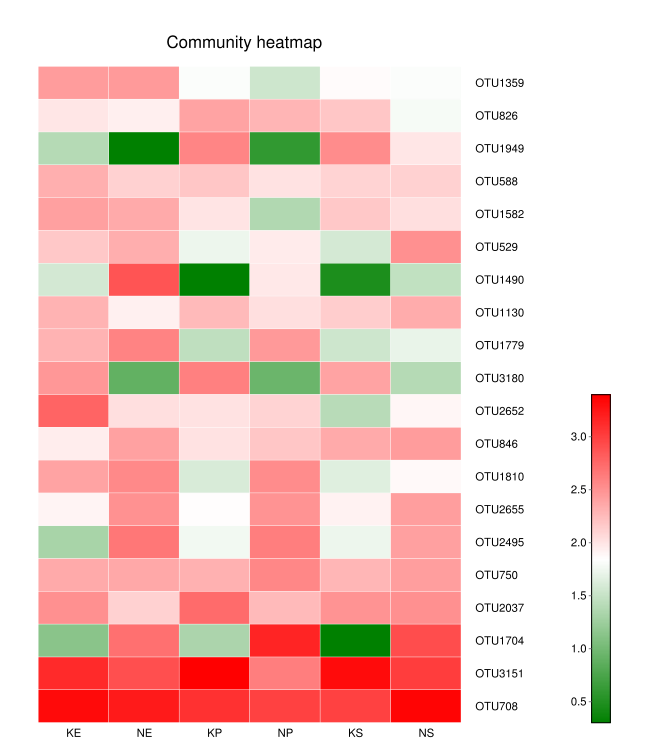


a


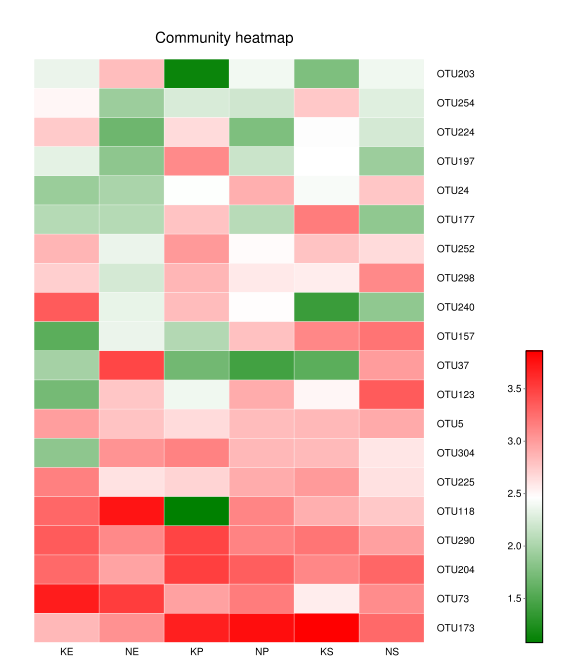


b

**Fig. S4.** Community heatmap showing top 30 microbial genus in all sample (a: bacteria; b: fungi).
